# Comparison of qPCR versus culture for the detection and quantification of *Clostridium difficile* environmental contamination

**DOI:** 10.1371/journal.pone.0201569

**Published:** 2018-08-30

**Authors:** Laura K. MacDougall, George Broukhanski, Andrew Simor, Jennie Johnstone, Samira Mubareka, Allison McGeer, Nick Daneman, Gary Garber, Kevin A. Brown

**Affiliations:** 1 Infection Prevention and Control, Public Health Ontario, Toronto, Ontario, Canada; 2 Sunnybrooke Health Sciences Centre, Toronto, Ontario, Canada; 3 Department of Medicine, University of Toronto, Toronto, Ontario, Canada; 4 Mt. Sinai Healthcare System, Toronto, Ontario, Canada; 5 Department of Medicine, University of Ottawa, Ottawa, Canada; 6 Dalla Lana School of Public Health, University of Toronto, Toronto, Ontario, Canada; Cornell University, UNITED STATES

## Abstract

Contaminated surfaces serve as an important reservoir for *Clostridium difficile* transmission. Current strategies to detect environmental contamination of *C*. *difficile* rely heavily on culture, and often only indicate presence versus absence of spores. The goal of this study was to compare quantitative PCR (qPCR) to culture for the detection and quantification of *C*. *difficile* from inert surfaces. First, we compared the limit of detection (LOD) of a 16S rRNA gene and toxin B gene qPCR assay for detection of *C*. *difficile* in solution. Second, we compared the LODs of 16S rRNA gene qPCR versus culture for detection of *C*. *difficile* from surfaces. Solution experiments were performed by direct seeding of spores into neutralizing broth, whereas surface experiments involved seeding of spores onto plastic test surfaces, and recovery using sponge swabs. Both experiments were conducted using spores expressing short (NAP1) and long (NAP4) hair lengths. Combining data from both strains, the overall LOD for *C*. *difficile* cells in solution was 1.4 cells for 16S rRNA gene and 23.6 cells for toxin B gene qPCR (p<0.001). The overall LOD for *C*. *difficile* cells from surfaces was 17.1 cells for 16S rRNA gene qPCR and 54.5 cells for culture (p = 0.05), and was not statistically different between strains for each method (p = 0.52). Overall, the proportion of *C*. *difficile* cells recovered from surfaces was good when detected by 16S rRNA gene qPCR and culture (qPCR: 76%, culture: 67%, p = 0.36), but, 16S rRNA gene qPCR was capable of detecting lower levels of surface contamination. Future work attempting to measure the presence of *C*. *difficile* on environmental surfaces should consider using qPCR.

## Introduction

*Clostridium difficile* is a Gram-positive, anaerobic, spore-forming bacterium, and the leading cause of health-care associated infective diarrhea [1]. Risk factors for the disease include previous hospitalizations, advanced age and the use of antibiotics [1–3]. *C*. *difficile* spores play an important role in disease transmission. Spores can persist in the environment for up to several months and are resistant to stresses such as heat, oxygen, and exposure to routine disinfectants [4,5].

Within the hospital environment, contaminated surfaces are thought to serve as an important reservoir for *C*. *difficile* transmission [6]. Unfortunately, the optimal method for sampling and detecting *C*. *difficile* contamination is currently unknown. Different sampling techniques have been reported, including contact plates, swabs, wipes and sponges, with several studies suggesting superior performance by sponges [7–9]. Culture remains the primary laboratory detection method, despite the routine and successful use of PCR in the clinical setting. Furthermore, most studies are qualitative and report only the presence or absence of *C*. *difficile* in small surface area samples [10,11]. Previous work has demonstrated that quantitative PCR (qPCR) can be successfully used for environmental sampling of *C*. *difficile*, but assay sensitivity and direct comparison to culture have not been evaluated [12].

Differences in the ability of *C*. *difficile* strains to adhere to surfaces may also be an important factor in disease transmission, and may impact the quantification of spores when conducting environmental sampling. Attachment and adherence of *C*. *difficile* spores is thought to be influenced by hydrophobicity, the exosporium and the presence of hair-like structures identified on the surface of certain strains [13–15]. Two epidemic strains with different surface properties are NAP1 and NAP4. NAP1 spores express short hairs, while NAP4 spores express long hairs [15,16].

Our objective was to validate qPCR as a sensitive method to quantify *C*. *difficile*, and compare qPCR to culture for detection *C*. *difficile* spores on contaminated surfaces. Furthermore, we compared both NAP1 and NAP4 spores to determine whether potential differences in adhesion affect the ability to quantify environmental contamination.

## Materials and methods

### Study design

Under experimental conditions, we compared qPCR versus quantitative culture for detection of *C*. *difficile* surface contamination. As a preliminary step, we assessed a toxin B gene and 16S rRNA gene qPCR assay and proceeded with the most sensitive target for *in vitro* studies. Test surfaces (plastic) were then seeded with known quantities of *C*. *difficile* spores (NAP1 and NAP4), and recovered using sponge swabs. Recovered spores were detected by qPCR and culture, and the recovery efficiencies (% recovery) and limit of detections (LODs) determined to establish the most sensitive method.

### Culture of *C*. *difficile* and preparation of spore stocks

All *C*. *difficile* cultures were grown under anaerobic conditions at 37°C. To induce sporulation, *C*. *difficile* was cultured using Brucella supplemented agar (BSA) for approximately 10 days. For all other quantitative culturing, *C*. *difficile* was grown on CHROMagar media (Alere Inc., Canada) for 24 h.

NAP1 and NAP4 spores were obtained from clinical specimens and typed at the Public Health Ontario Laboratories. To prepare NAP1 and NAP4 spore stocks, *C*. *difficile* was scraped from BSA plates and suspended in 1 mL of double distilled water (ddH_2_O). Suspensions were washed three times in 1 mL of ddH_2_O, incubated overnight at 4°C to kill the majority of vegetative cells, and then washed one additional time. Stock concentrations of roughly 1 x 10^7^ CFU/mL were prepared by adjusting the optical density (600 nm) of the suspension to 0.15. Actual concentrations were determined by cell counting using a disposable hemocytometer (In Cyto, Neubauer Improved, VWR, Canada) to provide the total number of cells, regardless of culturability. The morphology of the cells and absence of debris were evaluated by phase-contrast microscopy. Spore stocks were stored for approximately 1 week at 4°C and re-quantified before use to ensure accuracy.

### Detection of *C*. *difficile* by qPCR

Primer and probe sequences used in this study are listed in S1 Table. For qPCR, 10 μL reactions containing 5 μL JumpStart Taq ReadyMix for Quantitative PCR (D7440, Sigma, Canada), 0.1 μL Reference Dye for Quantitative PCR (R4526, Sigma, Canada), 0.4 μL 25mM MgCl_2_, 0.5 μM each of forward and reverse primers, 0.15 μM TaqMan probe and 3.85 μL DNA were prepared. Reactions were run in triplicate on an ABI 7900HT thermocycler (Applied Biosystems) under the following conditions: 50°C 2 min, 95°C 10 min, then 45 cycles of 95°C 15 sec, 60°C 1 min.

DNA was extracted from spore solutions using the ZymoBIOMICS DNA Miniprep Kit (Cedarlane, Canada) according to the manufacturer’s instructions with minor modifications: a bead-beating step of 1 h was used and DNA was eluted in a final volume of 50 μL of ddH_2_O. PCR efficiencies of the 16S rRNA gene and toxin B gene assays were determined by extraction of DNA from a known concentration of spores (OD = 0.15), followed by ten-fold serial dilution in nuclease-free water (10^−1^ to 10^−8^) to generate standards. Each ten-fold serial dilution was performed in triplicate and used for both the 16s rRNA gene and toxin B gene assays. Standard curves were constructed by plotting C_t_ values against log_10_ of the spore quantities corresponding to each DNA dilution. A linear regression line was fit to the data using least squares and PCR efficiency was calculated according to Eq 1.

$$\text{PCR Efficiency}=10^{-1/slope}-1$$ (1)

### LOD from solution

We considered the LOD from solution to be the minimum number of *C*. *difficile* cells in dilution to be detected in over 95% of experimental replicates when subjected to the entire process of DNA extraction and qPCR [17]. We determined LOD values based on the total number of cells in solution, which was obtained via counting using a haemocytometer.

Ten-fold serial dilutions of *C*. *difficile* cells were prepared from a quantified spore stock (OD = 0.15) in 20% D/E Neutralizing Broth (Scigene, Canada). DNA extractions were performed on each dilution to generate standards, and the 16s rRNA gene and toxin B gene qPCRs were run as described above. A logistic regression model with detection (a C_t_ value < 40) as the outcome, and log_10_ cell quantity as the only predictor variable, was used [17]. The model was fit with the rstanarm package in R which conducts Markov Chain Monte Carlo estimation. Based on this model, the number of cells needed to achieve 95% probability of detection was estimated, along with the 95% credible interval (CI).

### Seeding and recovery of *C*. *difficile* cells from test surfaces

Polypropylene plastic was chosen as a test surface because it widespread throughout hospital environments in North America. Plastic surfaces were seeded with known quantities of *C*. *difficile* by pipetting 10 μL drops of serially diluted cells onto marked 25-cm^2^ areas. Spiked surfaces were allowed to dry for 2 h at room temperature before sampling. Surfaces were sampled using sterile cellulose sponges, pre-moistened with D/E Neutralizing Buffer (Scigiene, Canada). The entire marked area was wiped horizontally, vertically and at a 45° angle; the whole process was repeated twice. Sponges were then dispensed into sampling bags and 40 mL of sterile ddH_2_O was added. Each sponge was massaged vigorously between finger tips for 90 seconds, and then allowed to stand at room temperature for 10 minutes. Sponges were aseptically removed and the liquid was transferred to a 50-mL conical tube. Extracted cells were collected by centrifugation at 7,500 × g for 15 minutes. All but 1 mL of the liquid was removed. The pelleted cells were re-suspended, transferred to a 1 mL microfuge tube, and then collected again by centrifugation at 10,000 × g for 5 min. The entire supernatant was decanted and the pellet was re-suspended in 500 μL of 20% D/E Neutralizing Broth. Half of the solution was used for DNA extraction and qPCR as described above. The remaining half was used for quantitative culture.

### LOD from surfaces

We defined the LOD from surfaces to be the minimum number of *C*. *difficile* cells seeded onto a surface that could be detected in over 95% of experimental replicates [17]. LODs were calculated using the logistic regression model described above and were based on the total number of cells seeded onto the surface.

### Mechanical recovery calculations

The mechanical recovery was used as measurement of the combined ability of the sponge to collect spores from a contaminated surface, and how well spores could be extracted from the sponge for quantification. The mechanical recovery by culture and qPCR were determined according to Eqs 2 and 3, respectively. Serial dilutions of a known concentration of *C*. *difficile* cells were prepared in water. Identical numbers of cells were seeded onto test surfaces and either directly onto culture plates for assessing the mechanical recovery by culture, or directly into 500 μL of 20% D/E Neutralizing Broth for assessing the mechanical recovery by qPCR. Cells were recovered from the surface and processed by quantitative culture or qPCR as described above. Graphs and statistical analyses were conducted using Graph Pad Prism 6.0

$$\text{Mechanical recovery by culture}=\frac{\text{CFU recovered from surface}}{\text{CFU in the seeded volume}}\times 100$$ (2)

$$\text{Mechanical recovery by qPCR}=\frac{\text{Number of cells recovered from surface by qPCR}}{\text{Number of cells in the seeded volume by qPCR}}\times 100$$ (3)

## Results

### Validation of the 16S rRNA gene and toxin B gene qPCR assays

The *C*. *difficile* 16S rRNA gene and toxin B gene qPCRs produced standard curves with PCR efficiencies between 93–100% (S1 Fig). PCR efficiencies were slightly lower for the toxin B gene assay (95.1 for NAP1 and 93.0% for NAP4) when compared to the 16S rRNA gene assay (99.5% for NAP1 and 99.9% for NAP4). All PCR efficiencies fell within the acceptable range of 90–110% [18].

### LOD from solution for the 16S rRNA gene and toxin B gene qPCR assays

**Fig 1:**
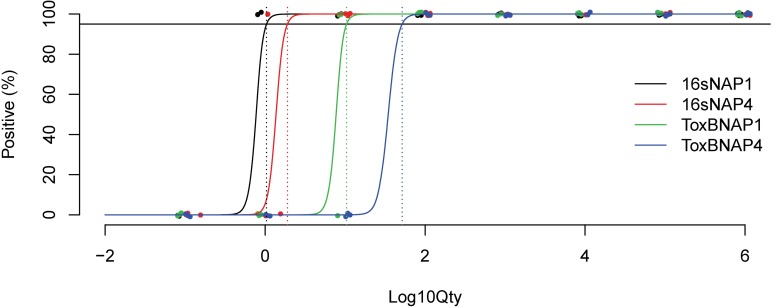
LOD curves for the *C*. *difficile* 16S rRNA gene and toxin B gene qPCR assays using NAP1 and NAP4 spores. LODs (dotted lines) were determined at the 95% positivity (solid black line) cut-off (n = 3 independent experiments for each strain/assay combination, total n = 12).

The LOD for the 16S rRNA gene qPCR was 16.9-fold lower than for the toxin B gene qPCR (1.4 for 16s rRNA versus 23.6 for toxin B, p<0.001, Table 1 and Fig 1). The LOD for NAP1 was 3.1-fold lower than NAP4 (3.3 for NAP1 versus 10.1 for NAP4, p<0.001). No statistically significant interaction between method and strain was detected in the model (p = 0.20).

**Table 1:** LOD of the 16S rRNA gene and toxin B gene qPCR assays recovery of *C*. *difficile* spores from solution.

PCR Target	Strain	n^a^	LOD (# cells, 95% CI)
16S rRNA gene	NAP1	3	1.0 (0.7–2.3)
	NAP4	3	1.9 (1.3–6.5)
	Overall	6	1.4 (1.1–3.4)
Toxin B gene	NAP1	3	10.4 (7.1–22.4)
	NAP4	3	52.4 (16.5–160.6)
	Overall	6	23.6 (12.5–52.2)

^a^n = number of independent experiments, 8 dilutions per experiment

### LOD from surfaces for 16S rRNA gene qPCR and culture

**Fig 2:**
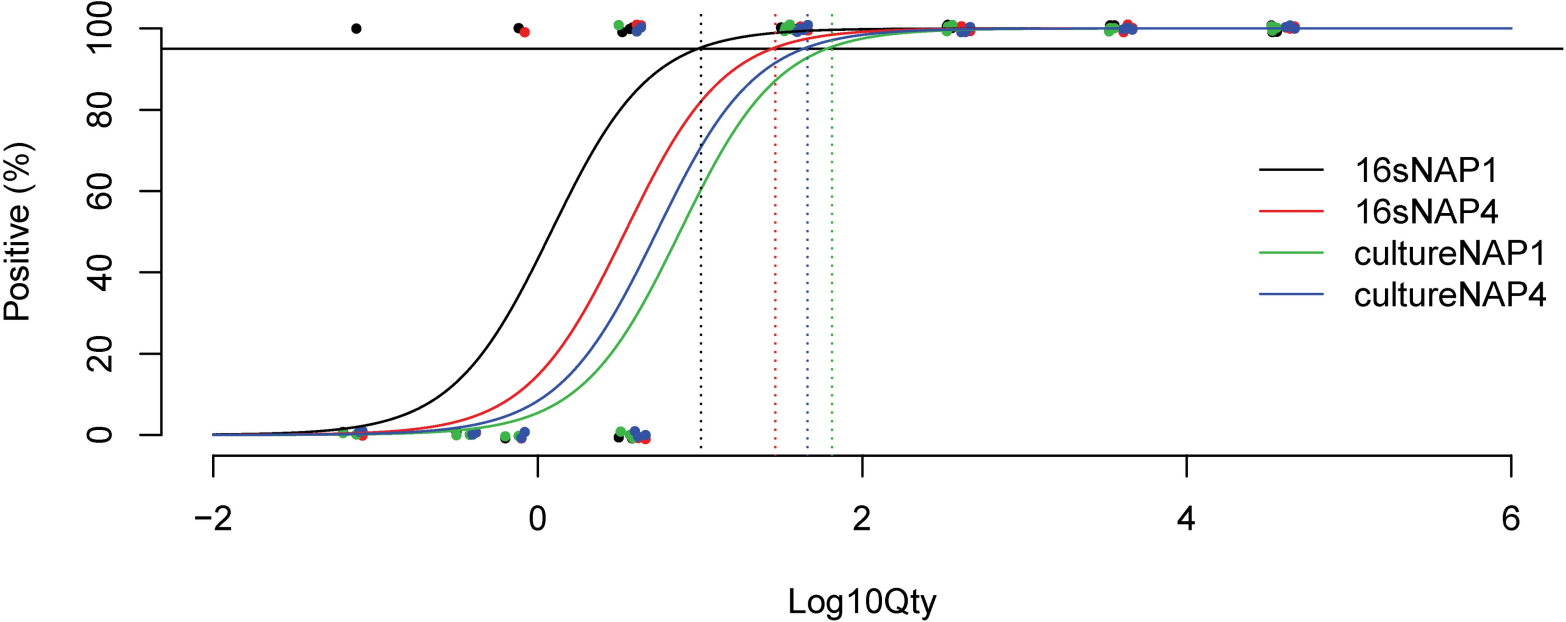
LOD curves for the recovery of NAP1 and NAP4 spores from polypropylene plastic using cellulose sponges by qPCR (16S rRNA gene) and culture. LODs (dotted lines) were determined at the 95% positivity (solid black line) cut-off (n = 5 independent experiments for each strain/assay combination, total n = 20).

Overall, the LOD for 16S rRNA gene qPCR was 3.2-fold lower than that for culture (17.1 for 16S rRNA gene qPCR versus 54.5 for culture, p = 0.05, Table 2 and Fig 2). No significant difference was found between the LODs for NAP1 versus NAP4 (p = 0.52). No statistically significant interaction between method and strain was detected in the LOD model (p = 0.23).

**Table 2:** LOD of qPCR and culture for the recovery of *C*. *difficile* from contaminated surfaces.

Method	Strain	n^a^	LOD (# cells, 95% CI)
16S rRNA gene qPCR	NAP1	5	10.1 (2.9–56.4)
	NAP4	5	29.1 (8.3–160.3)
	Overall	10	17.1 (6.2–75.8)
Culture	NAP1	5	65.0 (16.3–422.9)
	NAP4	5	46.0 (13.5–275.6)
	Overall	10	54.5 (18.0–263.7)

^a^n = number of independent experiments, 5 dilutions per experiment

### Mechanical recovery from sponges

Because the 16S rRNA gene qPCR assay had a substantially lower LOD than the toxin B gene assay in solution, we chose to proceed with determining the mechanical recovery of *C*. *difficile* from contaminated surfaces by 16S rRNA gene qPCR for comparison to culture. The mechanical recoveries by qPCR (76%) and culture (67%) were not significantly different (p = 0.36), and were similar for both the NAP1 and NAP4 strains (NAP1 = 75%, NAP4 = 77%, p = 0.87 for qPCR and NAP1 = 76%, NAP4 = 58%, p = 0.08 for culture). No statistically significant interaction between method and strain was detected for mechanical recovery (p = 0.89 for NAP1 and p = 0.20 for NAP4).

## Discussion

In this study, we compared the use of qPCR to culture for the detection and quantification of two epidemic strains of *C*. *difficile*, NAP1 and NAP4, from contaminated surfaces. We found that the LOD of 16S rRNA gene qPCR was 16.9-fold lower than toxin B gene qPCR, and that the LOD of *C*. *difficile* from a surface by 16S rRNA gene qPCR was 3.2-fold lower than culture.

We initially assessed the performance and sensitivity of a 16S rRNA gene and toxin B gene qPCR assay for detection of *C*. *difficile* in solution. We found that the LOD of the 16S rRNA gene qPCR was over 15 times lower, which may be partly due to the higher copy number of the 16s rRNA gene and greater stability and efficiency of the 16S rRNA gene qPCR. 16S rRNA gene qPCR, therefore, has greater potential to identify low-level contamination, with the caveat that strains may not be toxigenic and require additional testing to confirm clinical relevance. In the hospital setting, this may not pose a significant limitation since the majority of surface-isolated *C*. *difficile* has been found to carry the *tcdA* and *tcdB* genes [19,20].

Different groups have compared PCR to culture for clinical detection of *C*. *difficile* in stool samples. In antibiotic-associated diarrhea patients, quantification by 16S rRNA gene qPCR correlated well with culture, but qPCR was more sensitive and able to detect *C*. *difficile* in several culture-negative cases [21]. In a more recent analysis involving spiked stool samples, the LOD of traditional PCR analyzed by gel electrophoresis was 10-fold higher than culture [22]. Overall, we found the LOD for qPCR was 3.2-fold lower compared to culture for environmental detection of *C*. *difficile*. Both methods proved to be highly sensitive, capable of identifying less than 55 spores from plastic surfaces, but our data indicate that qPCR is more likely to detect low-level contamination. Assuming a density of 1 cell/25 cm^2^, this study predicts qPCR would detect *C*. *difficile* in 27% of 25 cm^2^ samples, or 72% of 100 cm^2^ samples, compared to only 7% and 34% by culture, respectively. Therefore, in practice, both switching to 16s rRNA gene qPCR and increasing the sample surface area are strategies that are likely to yield higher probability of detecting spores. Our findings are in agreement with a previous report that was unable to detect *C*. *difficile* from inert hospital surfaces by culture, but found up to 40% of samples positive by qPCR [23]. Improved environmental detection by qPCR over culture has been similarly reported for other bacterial species, including *Bacillus subtilus* and *Erwinia herbicola* [24,25].

We obtained relatively high mechanical recoveries from surfaces of approximately 70%, supporting efficient surface removal and subsequent extraction of spores from sponges reported by previous groups [7–9]. In similar validation experiments using various sampling methods, Ali *et al*. recovered 76–94% [7] and Claro *et al*. recovered 14–92% [26] of *C*. *difficile* from a variety of surface materials by culture. An interesting finding from this study was the lack of difference in the recovery of the *C*. *difficile* spores from strains with different surface properties. Here we compared NAP1 and NAP4, the two most dominant strains associated with hospital acquired infections in Canada [27]. We speculated that shorter hairs due to truncation of the *bclA* gene [16] may result in differences in adhesion. Our ability to recover spores of both types at similar efficiencies suggests similar adhesion between the two strains to plastic, but we cannot rule out the possibility of differences in adhesion to other surface materials or after longer periods post-deposition.

Our study had several limitations. First, we determined the LODs for the recovery of *C*. *difficile* from only a plastic surface material that is most widespread throughout hospital environments in North America. Previous studies have demonstrated that the recovery of *C*. *difficile* from inanimate surfaces varies according to material type [26], which may have changed results for the NAP1 versus NAP4 adhesion comparisons. Second, the LOD of qPCR is limited by the DNA extraction efficiency and the quantity of DNA used in the assay; we determined the LOD for qPCR using only a single DNA extraction kit. However, prior to conducting our study, we tested several DNA extraction methods and selected this technique since it returned the best yield (S2 Table). Third, we did not consider differences in the recovery or detection of spores versus vegetative cells, which are likely to exhibit differences in culturability and DNA extraction efficiency. However, spores are generally thought to represent the more important form of *C*. *difficile* from a transmission perspective, due to the rapid death of vegetative cells in the environment. Finally, our study design did not consider the effects of environmental cleaning or the duration of air exposure, both of which would be expected to reduce culturability. Our study used a short (<3 h) air exposure in order to minimize bias towards qPCR, which is capable of detecting both non-culturable but nevertheless viable cells, and non-viable cells.

In conclusion, few studies have exploited qPCR for detection of environmental *C*. *difficile* contamination. Our study demonstrates that 16S rRNA gene qPCR is substantially more sensitive than culture for the detection of environmental *C*. *difficile* contamination. The use of qPCR for detection of environmental *C*. *difficile* has the potential to offer a number of advantages over culturing including reduced cost and turnaround time for results, as well as the ability to detect both culturable and non-culturable contamination. Future studies quantifying the environmental burden of *C*. *difficile* should consider 16S rRNA gene qPCR for improved detection and enumeration.

## Supporting information

S1 Fig **(A-D) Standard curves corresponding to the 16S rRNA gene qPCR assays for NAP1 and NAP4 *C*. *difficile* strains.** DNA was extracted from known concentrations of *C*. *difficile* cells and serially diluted (10^−1^ to 10^−8^) to generate standards. C_t_ values produced by qPCR reactions for each standard were plotted against log_10_(cell quantity) and a linear curve was fit to the data (E = PCR efficiency).
(DOCX)

S1 Table Primer and probe sequences used in this study. (DOCX)

S2 Table DNA extraction methods and corresponding yields tested prior to initiation of the study. NAP1 and NAP4 *C*. *difficile* spore solutions of roughly 1 x 10^7^ CFU/mL were tested.
(DOCX)

S3 Table Raw data used to produce Fig 1 and Table 1. (CSV)

S4 Table Raw data used to produce Fig 2 and Table 2. (CSV)

S5 Table Raw data used to produce S1 Fig. (XLSX)
