# Supplementary material for: Comparison of qPCR versus culture for the detection and quantification of Clostridium difficile environmental contamination
Source: PLoS One. 2018 Aug 30;13(8):e0201569. doi: 10.1371/journal.pone.0201569 (PMC6116935; doi:10.1371/journal.pone.0201569)
Supplement: S1 Fig — (A-D) Standard curves corresponding to the 16S rRNA gene qPCR assays for NAP1 and NAP4 C. difficile strains. DNA was extracted from known concentrations of C. difficile cells and serially diluted (10−1 to 10−8) to generate standards. Ct values produced by qPCR reactions for each standard were plotted against log10(cell quantity) and a linear curve was fit to the data (E = PCR efficiency). (DOCX) [file pone.0201569.s001.docx]

**Supporting information:**

**S1 Fig**. **(A-D) Standard curves corresponding to the 16s rRNA qPCR assays for NAP1 and NAP4 *C. difficile* strains.** DNA was extracted from known concentrations of *C. difficile* cells and serially diluted (10^-1^ to 10^-8^) to generate standards. C_t_ values produced by qPCR reactions for each standard were plotted against log_10_(cell quantity) and a linear curve was fit to the data (E = PCR efficiency).
