# Supplementary material for: Comparison of qPCR versus culture for the detection and quantification of Clostridium difficile environmental contamination
Source: PLoS One. 2018 Aug 30;13(8):e0201569. doi: 10.1371/journal.pone.0201569 (PMC6116935; doi:10.1371/journal.pone.0201569)
Supplement: S1 Table — (DOCX) [file pone.0201569.s002.docx]

**Supporting information:**

**S1 Table. Primer and probe sequences used in this study.**

| **Name** | **Sequence (5`- 3`)** | **Reference** |
| --- | --- | --- |
| 16s_Fwd | TTGAGCGATTTACTTCGGTAAAGA | [1] |
| 16s_Rev | CCATCCTGTACTGGCTCACCT | [1] |
| 16s_Probe | 6-FAM-CGGCGGACGGGTGAGTAACG-MBG | [1] |
| ToxB_Fwd | GAAGGATTACCTGTAATTGC | [2] |
| ToxB_Rev | CTGCCATTATACCTATCTTAGC | [2] |
| ToxB_Probe | JOE-CTCTTTGAT-ZEN-TGCTGCACCTAAACTTACACC-Iowa Black FQ | [2] |

**References:**

1. Mutters R, Nonnenmacher C, Susin C, Albrecht U, Kropatsch R, Schumacher S. Quantitative detection of Clostridium difficile in hospital environmental samples by real-time polymerase chain reaction. J Hosp Infect. 2009;71: 43–48. doi:10.1016/j.jhin.2008.10.021

2. Kilic A, Alam MJ, Tisdel NL, Shah DN, Yapar M, Lasco TM, et al. Multiplex Real-Time PCR Method for Simultaneous Identification and Toxigenic Type Characterization of *Clostridium difficile* From Stool Samples. Ann Lab Med. 2015;35: 306. doi:10.3343/alm.2015.35.3.306
