# Supplementary material for: Comparison of qPCR versus culture for the detection and quantification of Clostridium difficile environmental contamination
Source: PLoS One. 2018 Aug 30;13(8):e0201569. doi: 10.1371/journal.pone.0201569 (PMC6116935; doi:10.1371/journal.pone.0201569)
Supplement: S2 Table — NAP1 and NAP4 C. difficile spore solutions of roughly 1 x 10^7^ CFU/mL were tested. (DOCX) [file pone.0201569.s003.docx]

**Supporting information:**

**S2 Table. DNA extraction methods and corresponding yields tested prior to initiation of the study.** NAP1 and NAP4 *C. difficile* spore solutions of roughly 1 x 10^7^ CFU/mL were tested.

| **Method** | **Typical DNA Yield (ng/μL)** |
| --- | --- |
| Qiagen QIAamp DNA Stool Mini Kit | 0.5 – 1 |
| Silicon Bead Beating with Qiagen QIAamp DNA Stool Mini Kit | 0 - 1 |
| MoBio Powersoil DNA Isolation Kit | 8 |
| ZymoBIOMICS DNA Miniprep Kit | 15 - 20 |
